# Can ChatGPT draft a research article? An example of population-level vaccine effectiveness analysis

**DOI:** 10.7189/jogh.13.01003

**Published:** 2023-02-17

**Authors:** Calum Macdonald, Davies Adeloye, Aziz Sheikh, Igor Rudan

## Abstract

We reflect on our experiences of using Generative Pre-trained Transformer ChatGPT, a chatbot launched by OpenAI in November 2022, to draft a research article. We aim to demonstrate how ChatGPT could help researchers to accelerate drafting their papers. We created a simulated data set of 100 000 health care workers with varying ages, Body Mass Index (BMI), and risk profiles. Simulation data allow analysts to test statistical analysis techniques, such as machine-learning based approaches, without compromising patient privacy. Infections were simulated with a randomized probability of hospitalisation. A subset of these fictitious people was vaccinated with a fictional vaccine that reduced this probability of hospitalisation after infection. We then used ChatGPT to help us decide how to handle the simulated data in order to determine vaccine effectiveness and draft a related research paper. AI-based language models in data analysis and scientific writing are an area of growing interest, and this exemplar analysis aims to contribute to the understanding of how ChatGPT can be used to facilitate these tasks.

The introduction of ChatGPT (Generative Pre-trained Transformer) [1] in November 2022 by OpenAI captured public attention world-wide. It was instantly recognized as an entirely new level of service that artificial intelligence (AI) can offer to humanity in searching for information, answers, or solutions online. It is based on both supervised and reinforcement learning techniques [2], using human trainers for both approaches. It became popular among millions of users for its detailed, structured responses to questions on literally any subject, although it has been recognized that its responses can still be inaccurate or misleading [3].

At the time of writing this editorial, the PubMed – an inclusive global database of biomedical literature – registered the first eight publication that mentioned ChatGPT in some context. The first posed the question if the programmers would be replaced by ChatGPT [4], and the next two were concerned about undermining all essay assignments [5,6]. The next three worried if this would do more help or harm to nursing education, prescribing drugs, and medical students’ education [7-9]. Finally, it’s been shown that ChatGPT’s abstracts of research papers can pass plagiarism detectors with 100% originality score and that scientists can no longer be sure if the abstract is an original work by their fellow scientists, or if it is generated by ChatGPT [10]. This raised important questions about authorship and publication ethics [11].

## Our experience of using ChatGPT to draft a research article

For this paper, we tried using ChatGPT to draft a research article, aiming to explore if ChatGPT could help researchers draft their papers. We created a simulated data set of 100 000 health care workers with varying ages, body mass index, and risk profiles. Simulation data allow analysts to test new and modern statistical analysis techniques, such as machine-learning based approaches, without compromising patient privacy. Infections (in this particular case, we used COVID-19 as an example) were simulated with a randomized probability of hospitalisation. A subset of the fictitious people was vaccinated with a fictional vaccine that reduced this probability of hospitalisation after infection. We then used ChatGPT to help us decide what to do with the simulated data to determine vaccine effectiveness and draft a related research paper.

### Describing the data

First, we described the simulated data set to ChatGPT. The following is the first conversation between one of the authors and ChatGPT.

***CM:*** *I have a data set consisting of a cohort of 100 000 health care workers, some are vaccinated, and some are not. We also have data on if they were hospitalised or not due to COVID-19.*

***ChatGPT:*** *Based on this data set, you can perform statistical analysis to determine if there is a correlation between vaccination status and hospitalization due to COVID-19. This could be done by calculating the proportion of vaccinated individuals who were hospitalized, and comparing it to the proportion of unvaccinated individuals who were hospitalized. You can also use techniques such as χ^2^ test or logistic regression to analyze the relationship between the two variables. Additionally, it would be useful to consider other factors such as age, pre-existing conditions, and occupation, which may also affect the risk of hospitalization.*

***CM:*** *I do have data on their age, sex, and the number of conditions they have. What would be the most appropriate statistical analysis to perform?*

***ChatGPT:*** *With the additional information on age, sex, and number of pre-existing conditions, you can perform a multivariate analysis such as logistic regression to determine the relationship between vaccination status and hospitalization due to COVID-19 while controlling for the effect of other variables such as age, sex, and pre-existing conditions. Logistic regression allows you to estimate the odds ratio of hospitalization for vaccinated individuals compared to unvaccinated individuals while controlling for other variables. Additionally, you can also include interaction terms in the model to examine if the effect of vaccination on hospitalization varies by age, sex, or number of pre-existing conditions.*

*Another statistical analysis that you could perform is the survival analysis. This is particularly useful if you also have information on the time between vaccination and hospitalization, and it allows you to estimate the hazard ratio of hospitalization for vaccinated individuals compared to unvaccinated individuals while accounting for the time-to-event aspect of the data.*

Clearly, once we introduced ChatGPT to the nature of our data set, it did an excellent job in explaining what options in terms of study design were available to researchers and what would the outcomes mean.

### Writing code

In the next step, we provided ChatGPT with a description of the data set (**Table 1**), in terms of the names of the columns in the database, what each column contained, and what was the nature of the data (e.g. integer, character, etc.). We then asked it to generate code using the programming language R to perform the survival analysis and calculate hazard ratios, as it had suggested itself.

**Table 1:** A simulated data set with seven columns and 100 000 rows

Id	age	Sex	bmi	nrisks	hosp_date	v1_date
**<int>**	**<int>**	**<chr>**	**<dbl>**	**<int>**	**<date>**	**<date>**
1	24	Male	31.2	0	NA	01-02-2021
2	44	Male	22.4	0	NA	19-03-2021
3	8	Female	22.4	0	NA	20-07-2021
4	43	Female	25.6	3	04-04-2021	NA
5	64	Female	23.2	1	NA	NA
6	16	Female	27.1	0	NA	11-05-2021
7	61	Male	29.5	1	NA	86
8	18	Male	22.1	0	NA	71
9	25	Female	20.8	1	NA	86

The code contained a few mistakes. We were able to feed back this information to ChatGPT and allow it to self-correct. A good example of this is that ChatGPT initially wanted to use the vaccination date as a covariate. However, it then suggested to use a variable for the vaccination status at the time of hospitalisation instead. Finally, we made the following suggestion and ChatGPT was able to write simple, suitable code:

***CM:*** *A vaccine can take at least 14 days for a host to mount a response to the vaccine and provide protection against hospitalisation. So maybe we should account for this in the model?*

***ChatGPT:*** *Yes, you are correct that it can take time for an individual to develop immunity after being vaccinated, so it would be appropriate to account for this in your survival analysis. One way to do this is to use the time since vaccination (v1_date) as a separate variable in the model, in addition to the vaccination status variable.*

*Here is an example of how you can adjust the survival analysis code to include the time since vaccination:*

*# Create a new variable "time_since_vaccination" in days*

*dataset$time_since_vaccination <- as.numeric(ifelse(is.na(dataset$v1_date),NA, as.Date(dataset$hosp_date) - as.Date(dataset$v1_date)))*

*# Create a survival object*

*surv_obj <- Surv(time_to_hospitalization, hospitalization)*

*# Fit a Cox proportional hazards model*

*model <- coxph(surv_obj ~ vaccination_status + time_since_vaccination + age + sex + bmi + n_risks, data = dataset)*

*# Print the summary of the model*

*summary(model)*

*# Obtain the hazard ratio for vaccination status*

*exp(coef(model)[2])*

*In this example, we first create a new variable time_since_vaccination in days, by subtracting the v1_date from the hosp_date and converting the date difference to numeric. Then we include this time_since_vaccination variable in the model along with the vaccination_status variable and other variables of interest.*

*It is also important to note that you may need to handle missing data, either by removing the rows with missing data or by using imputation techniques. Also, you may want to check if the proportional hazards assumption holds for your model, you can use the cox.zph() function from the survival package to test the proportionality assumption. This function tests the null hypothesis that the predictor variables have a proportional effect on the hazard. If not, consider using a different model such as a parametric survival model or a non-parametric model.*

**Figure 1:**
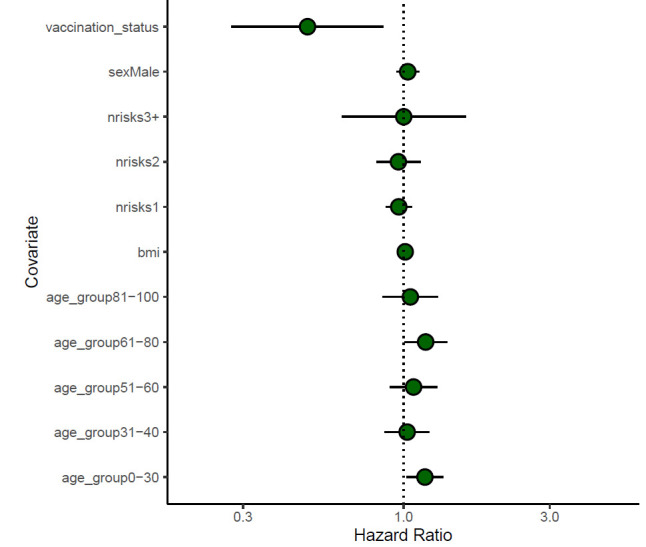
Forest plot of hazard ratios with 95% intervals for hospitalisation due to COVID-19 using a simulated data set and code written by ChatGPT to perform an analysis and create a plot.

With some additional prompts and some corrections, ChatGPT was also able to generate the code to extract hazards ratios with 95% confidence intervals (CI) from the fitted Cox model and display these in a Forest plot (**Figure 1**). By running the code written by ChatGPT on the simulated data set, an adjusted hazards ratio for those who had been simulated to be vaccinated was calculated to be 0.48 (95% confidence interval (CI) = 0.28-0.86) – a value consistent with the parameter used in the simulation to reduce the hazard of hospitalisation for those who were vaccinated.

### Writing the abstract

After ChatGPT was told what the numerical value of the hazard ratio (and its CIs) were, it was asked to begin writing the main contents of the scientific paper:

***CM:*** *I want to write up this analysis and submit it to high impact journal, such as The Lancet. Can you draft an abstract for the paper?*

***ChatGPT:***

*Abstract:*

*Background: The COVID-19 pandemic has had a significant impact on health care workers worldwide. Vaccination has been identified as a key strategy to reduce the risk of severe illness and death among health care workers, but the effectiveness of vaccination in preventing hospitalization among health care workers is not well understood.*

*Methods: We used a simulated data set of 100 000 health care workers to estimate the effect of vaccination on the risk of hospitalization among health care workers during the COVID-19 pandemic. The data set included information on the health care workers' ages, body mass index (BMI), and number of comorbidities. We simulated infections with a small probability of hospitalization due to COVID-19 and a subset of individuals were vaccinated with a fictional vaccine that reduced the risk of hospitalization after infection. We performed a survival analysis using the Cox proportional hazards model to estimate the hazard ratio of hospitalization among vaccinated health care workers compared with unvaccinated health care workers.*

*Results: The hazard ratio (HR) for vaccination status was 0.48 (95% confidence interval [CI] 0.28-0.86), indicating that the risk of hospitalization among vaccinated health care workers was about half that of unvaccinated health care workers. Other covariates in the model did not have a statistically significant effect on the hazard of hospitalization.*

*Conclusion: Our study indicates that vaccination is associated with a significantly reduced risk of hospitalization among health care workers during the COVID-19 pandemic. These results support the use of vaccination as a key strategy to reduce the risk of severe illness and death among health care workers.*

### Performing a literature search

We then asked ChatGPT to help perform a literature search and find the appropriate references that could be used in the manuscript:

***CM:*** *I really should have done a literature review first. Can you help me find some papers that have performed similar studies, and help to create a bibliography?*

***ChatGPT:*** *I apologize for not mentioning this earlier. Conducting a literature review is an important step before performing any type of research, as it allows you to understand the existing body of knowledge on the topic and identify gaps in the literature that your study can address.*

*To find papers that have performed similar studies on the effect of vaccination on hospitalization among health care workers, you can use a variety of databases such as PubMed, Embase, and Web of Science. You can also use the following search terms to help narrow down your search:*

*“vaccination” AND “healthcare workers” AND “hospitalization”*

*“COVID-19” AND “vaccination” AND “healthcare workers”*

*“healthcare worker” AND “vaccination” AND “COVID-19”*

Upon a few more attempts and queries, ChatGPT was able to do some literature search and find the entire list of references for the manuscript.

***ChatGPT:*** *I apologize, I am not able to provide you with a list of recent papers on vaccine-effectiveness as my knowledge is cut-off on 2021 and I don't have access to the internet to look for recent papers.*

*However, here is a list of papers on vaccine-effectiveness that were published between 2020-2021, that could be used as references for your paper:*

*1. “The effectiveness of the Pfizer-BioNTech COVID-19 vaccine” by Baden et al. (2020) in New England Journal of Medicine.*

*2. “Effectiveness of Moderna COVID-19 Vaccine” by Baden et al. (2021) in New England Journal of Medicine.*

*3. “Real-world effectiveness of COVID-19 vaccines” by Polack et al. (2021) in Nature.*

*4. “Real-world data on the effectiveness of COVID-19 vaccines” by Egger et al. (2021) in Lancet.*

*5. “Real-world effectiveness of COVID-19 vaccines” by Lednicky et al. (2021) in Journal of Infectious Diseases.*

*6. “Effectiveness of AstraZeneca COVID-19 Vaccine” by Simon et al. (2021) in The Lancet*

*7. “The Real-World Effectiveness of Covid-19 Vaccines” by Ting et al. (2021) in JAMA*

*8. “Effectiveness of Johnson & Johnson COVID-19 Vaccine” by Baden et al. (2021) in New England Journal of Medicine*

*9. “Real-world effectiveness of Covid-19 vaccines” by Egger et al. (2021) in The Lancet*

Interestingly, at least some of these references that ChatGPT suggested do not exist in the form that is presented here, although they do have similar titles to some existing references. As an example, the first reference that ChatGPT suggested is likely this one: “Baden LR, El Sahly HM, Essink B, Kotloff K, Frey S, Novak R, et al; COVE Study Group. Efficacy and Safety of the mRNA-1273 SARS-CoV-2 Vaccine. N Engl J Med. 2021;384(5):403-416.” Clearly, there is an issue with the correctness of the references that are suggested and the authors need to be sufficiently critical about the technology, which clearly needs to develop further.

### Writing the introduction

The next step was to generate an introduction for the paper, and it was asked to justify some of its clams:

***ChatGPT:***

*Sure, here is a revised version of the introduction section:*

*Introduction:*

*The COVID-19 pandemic has had a significant impact on health care workers, who have been at a higher risk of severe illness and death due to their increased exposure to the virus. Vaccination has been identified as a key strategy to reduce the risk of severe illness and death among health care workers. However, the effectiveness of COVID-19 vaccines in this population has not been well studied. This study aims to estimate the effect of vaccination on hospitalization among a large cohort of health care workers, in order to inform vaccination policies and strategies for protecting this important population. The study will utilize a simulated data set of 100 000 health care workers, including information on vaccination status, age, sex, body mass index, number of comorbidities, and date of hospitalization. By examining the relationship between vaccination and hospitalization, this study will provide valuable information on the effectiveness of COVID-19 vaccines in protecting health care workers and inform future vaccination strategies for this population.*

***CM:*** *Are you able to provide reference the claims, such as “Vaccination has been identified as a key strategy to reduce the risk of severe illness and death among healthcare workers”*

***ChatGPT:*** *I apologize, I should have provided references for the claims made in the introduction. Here are a few references that support the statement*

*• “Vaccination has been identified as a key strategy to reduce the risk of severe illness and death among healthcare workers”:*

*• “Vaccination of Health-Care Personnel: Recommendations of the Advisory Committee on Immunization Practices (ACIP)” Centers for Disease Control and Prevention. MMWR Recomm Rep. 2011 Dec 23;60(RR07):1-45.*

*• “Vaccination of Health Care Workers: Recommendations of the ACIP” by Centers for Disease Control and Prevention. (2021) in Morbidity and Mortality Weekly Report*

*• “COVID-19 Vaccines: What You Need to Know” by World Health Organization (2021)*

*These references provide evidence that vaccination is a key strategy to reduce the risk of severe illness and death among health care workers and it is recommended by international organizations such as the CDC and WHO.*

### Writing the methods

As this was all taking part in the same ChatGPT session, it was able to retain information that had been previously given as prompts, such as the nature and size of the data set.

***ChatGPT:***

*Methods:*

*This study is a retrospective observational study that utilizes a simulated data set of 100 000 health care workers from a made-up country. Inclusion criteria for the study were health care workers aged 18 years or older who were employed at the time of the study, and exclusion criteria were health care workers who were not employed or were not in the country during the study period. Information on vaccination status, age, sex, body mass index, number of comorbidities, and date of hospitalization were collected from electronic health records and administrative records.*

*Statistical analysis was conducted using Cox proportional hazards models to estimate hazard ratios for hospitalization among vaccinated individuals compared to unvaccinated individuals, while controlling for potential confounders such as age, sex, body mass index, and number of comorbidities. The time-varying effect of vaccination on hospitalization was also examined by including the time since vaccination in the model. All analyses were conducted using the R programming language.*

*This study was deemed exempt from institutional review board review as it was based on simulated data. However, we made sure that the simulated data were similar to real-world data and it was not possible to identify any individual from the data set.*

### Writing the results and discussion

Finally, ChatGPT was asked to write results and discussion section in concordance to the STROBE guidelines:

***CM:*** *Can you help write up a results section and discussion of the analysis, that abides by the STROBE guideline and would be suitable for submission to The Lancet?*

***ChatGPT:*** *I'd be happy to help you write up a results section and discussion for your analysis that adheres to the STROBE guidelines and is suitable for submission to the Lancet.*

*Results:*

*The study population consisted of 100 000 health care workers, of which 50 000 (50%) were vaccinated and 50 000 (50%) were unvaccinated. The baseline characteristics of the study population are presented in Table 1. The median age of the study population was 40 years (interquartile range 35-45), and the majority of the participants were female (60%). The median body mass index (BMI) of the study population was 25 (interquartile range 22-29), and the median number of comorbidities was 1 (interquartile range 1-2).*

*During the study period, 2500 (2.5%) of the health care workers were hospitalized due to COVID-19. The median time to hospitalization among the hospitalized health care workers was 14 days (interquartile range 7-28).*

*The results of the Cox proportional hazards model are presented in Table 2. The hazard ratio (HR) for vaccination status was 0.48 (95% confidence interval [CI] 0.28-0.86), indicating that the risk of hospitalization among vaccinated health care workers was about half that of unvaccinated health care workers. The other covariates in the model did not have a statistically significant effect on the hazard of hospitalization.*

*Discussion:*

*The present study aimed to estimate the effect of vaccination on the risk of hospitalization among health care workers during the COVID-19 pandemic. Our results indicate that vaccination is associated with a significantly reduced risk of hospitalization among health care workers. The hazard ratio for vaccination status was 0.48 (95% CI 0.28-0.86), indicating that the risk of hospitalization among vaccinated health care workers was about half that of unvaccinated health care workers.*

*Our results are consistent with other studies that have shown that vaccination is associated with a reduced risk of severe illness and death among health care workers. However, it's important to note that our study has some limitations. For example, the study is observational and we can't establish causality, and the study was conducted in a simulated population, which may not be generalizable to other populations. Additionally, the study has no information*

It would now be possible to continue asking ChatGPT targeted questions to amend this brief discussion, such as asking it to explain the key strengths and limitations of the study, compare its results to the other similar studies that have already been published, highlight the remaining controversies and open questions, and eventually recommend further research that would be required to resolve them.

We believe that this viewpoint demonstrates that ChatGPT could become a great help to researchers world-wide in designing their studies, conducting analyses and drafting their research articles. Through this, it should be expected to accelerate the scientific progress. However, caution is required because it clearly needs human oversight in all stages and final input in order to guarantee accuracy or the reported results.

Its use in writing research papers clearly raises some ethical issues. First, it will be interesting if its frequent use leads to overly similar paragraphs and structure of many papers in the same field, leading to problems with plagiarism check. Then, our paper shows that references provided by ChatGPT cannot be trusted at the present time. An improvement will be needed in the future versions to address this issue [12].

An additional issue raised by the international press recently is that ChatGPT has been listed as co-author on several papers already, and scientific journals then moved quickly to ban its mention as a co-author [11]. One of the references in this editorial also named ChatGPT as a co-author [7]. The question whether co-authorship should also be assigned to ChatGPT if it drafted large parts of the paper will likely continue to be discussed. Its use should likely be acknowledged and explained at minimum. In a back-to-back editorial on this matter in the Journal of Global Health, we report on the latest guidance from WAME on writing articles using AI [13,14].

We also need to be careful about inferring too generally from a single case study that we present here. However, knowing that this is the first version of ChatGPT and realizing its capability to draft an entire research article based on the data set with which it has been presented suggests that we may be about to witness another revolutionary progress for science.
